# Supplementary material for: Breaking through water-splitting bottlenecks over carbon nitride with fluorination
Source: Nat Commun. 2022 Nov 16;13:6999. doi: 10.1038/s41467-022-34848-8 (PMC9668818; doi:10.1038/s41467-022-34848-8)
Supplement: Supplementary file 1 — Supplementary Information [file 41467_2022_34848_MOESM1_ESM.pdf]

Supplementary Information

Breaking Through Water-Splitting Bottlenecks Over Carbon Nitride with Fluorination

Ji Wu[1], Zhonghuan Liu[1], Xinyu Lin[1], Enhui Jiang[1], Shuai Zhang[1], Pengwei Huo[1], Yan Yan[1]*, Peng Zhou[2]*, Yongsheng Yan[1]

[1]*Institute of Green Chemistry and Chemical Technology, School of Chemistry and Chemical Engineering, Jiangsu University, Zhenjiang 212013, PR China,*

[2]*Department of Electrical Engineering and Computer Science, University of Michigan, Ann Arbor, MI 48109, USA.*

Corresponding authors:

Y. Yan (dgy5212004@163.com)

P. Zhou (dpzhou@umich.edu)

Supplementary Figures

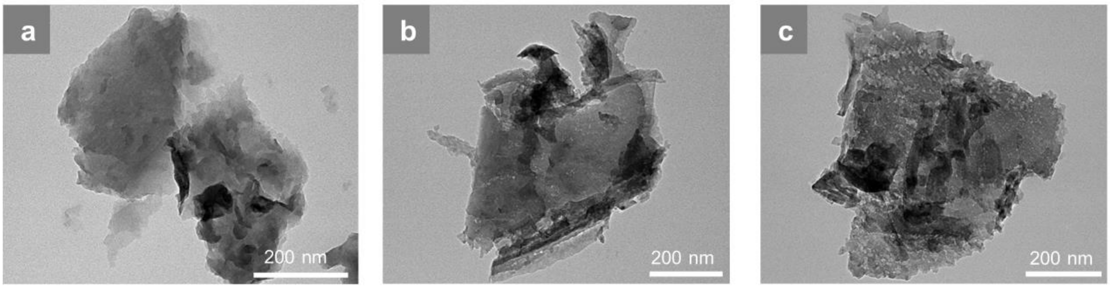

Supplementary Figure 1. Comparison of sample morphologies. TEM images of **(a)** CN, **(b)** CN-E and **(c)** $F_{0.1}$-CN.

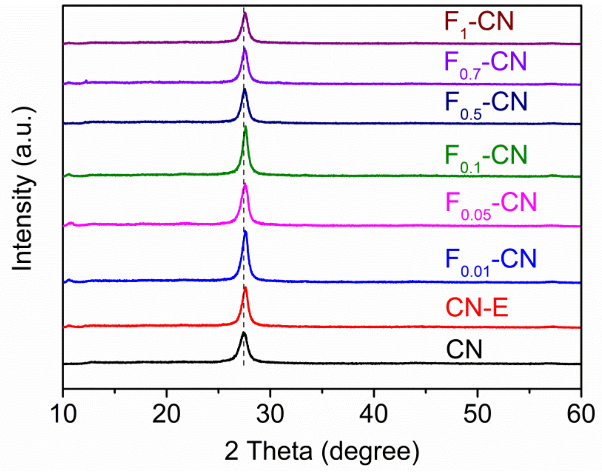

Supplementary Figure 2. Comparison of sample crystalline structure. XRD patterns of CN, CN-E and different F-CN catalysts.

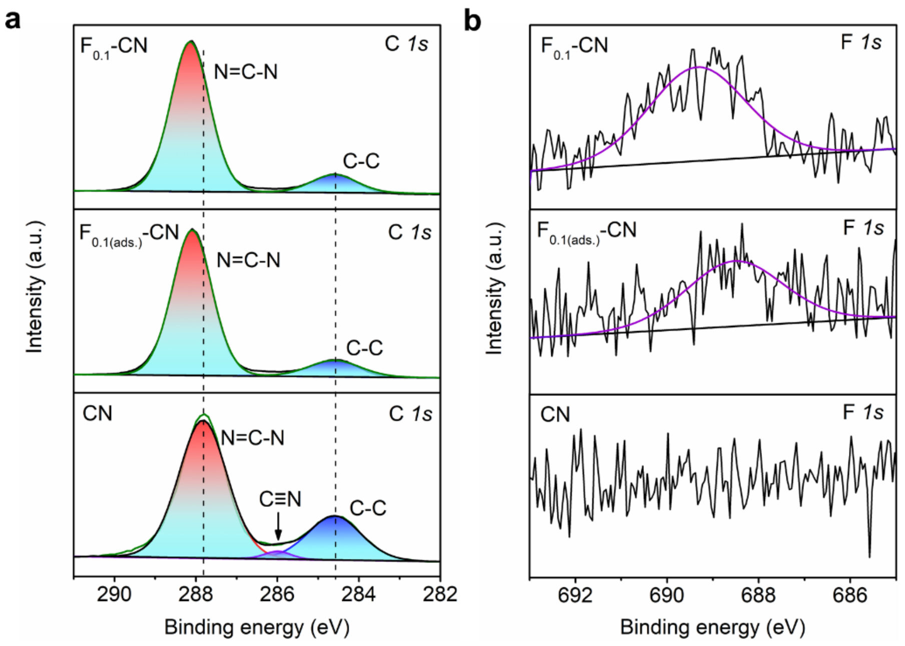

Supplementary Figure 3. Control experiments show C-F interaction. XPS **(a)** C1s and **(b)** F1s spectra of CN and F-CN samples before and after the hydrothermal treatment.

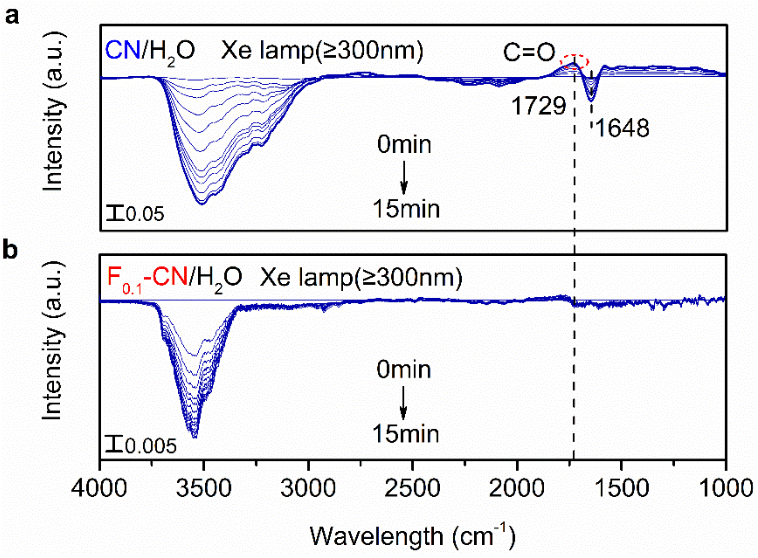

Supplementary Figure 4. Control experiments show the effect of light sources. DRIFTS spectra in-situ monitored at **(a)** CN/H_2O and **(b)** $F_{0.1}$-CN/ H_2O interface under constant white light (Xe lamp, ≥300 nm) irradiation in 15 min.

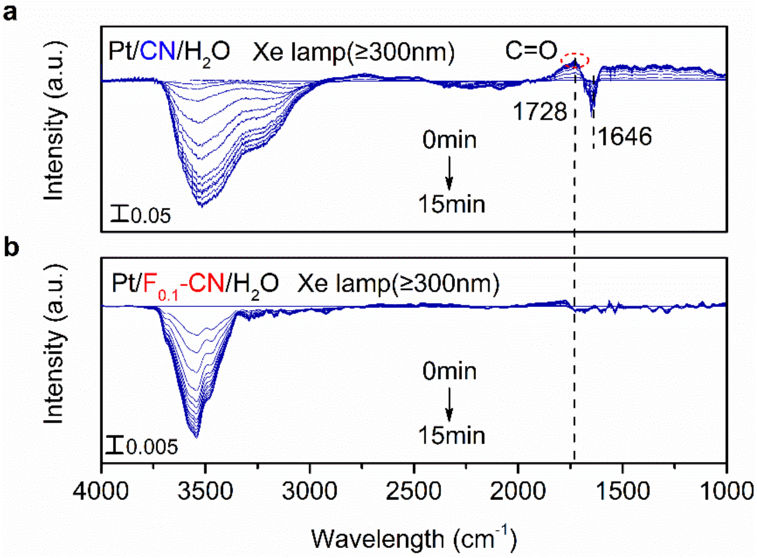

Supplementary Figure 5. Control experiments show the effect of Pt loading. DRIFTS spectra in-situ monitored at **(a)** Pt/CN/H_2O and **(b)** Pt/$F_{0.1}$-CN/ H_2O interface under constant white light (Xe lamp, ≥300 nm) irradiation in 15 min.

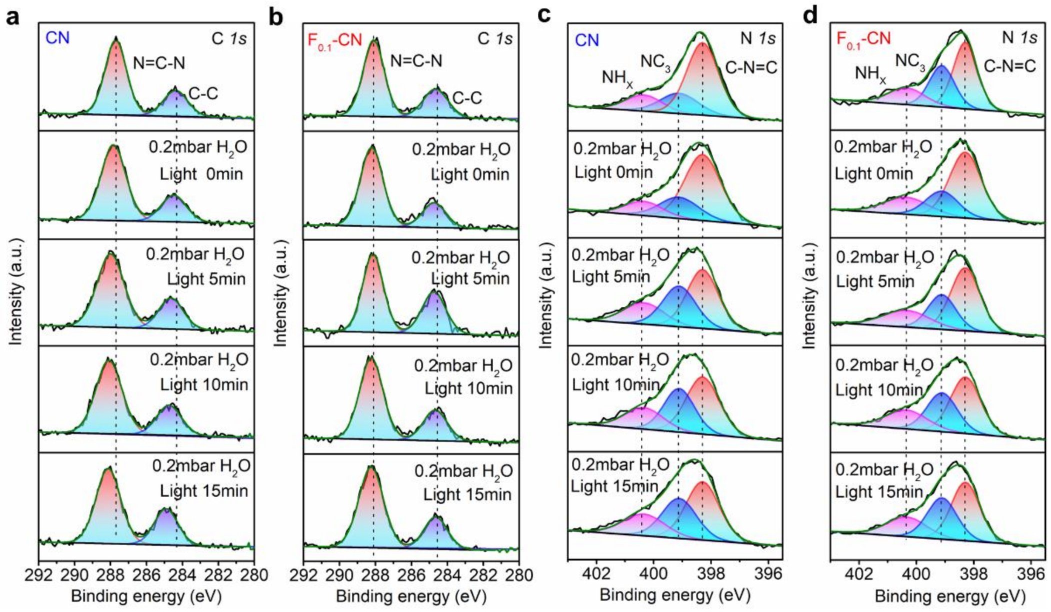

Supplementary Figure 6. Control experiments show changes in C*1s* and N*1s* spectra. In-situ NAP-XPS observations of C1s spectra on **(a)** pristine CN and **(b)** $F_{0.1}$-CN catalysts with 0.2 mbar H_2O vapor pressure using a 300 W Xenon lamp as the white light source in 15 min; and in-situ NAP-XPS observation of N*1s* spectra on **(c)** pristine CN and **(d)** $F_{0.1}$-CN catalysts with 0.2 mbar H_2O vapor pressure using a 300 W Xenon lamp as the white light source in 15 min.

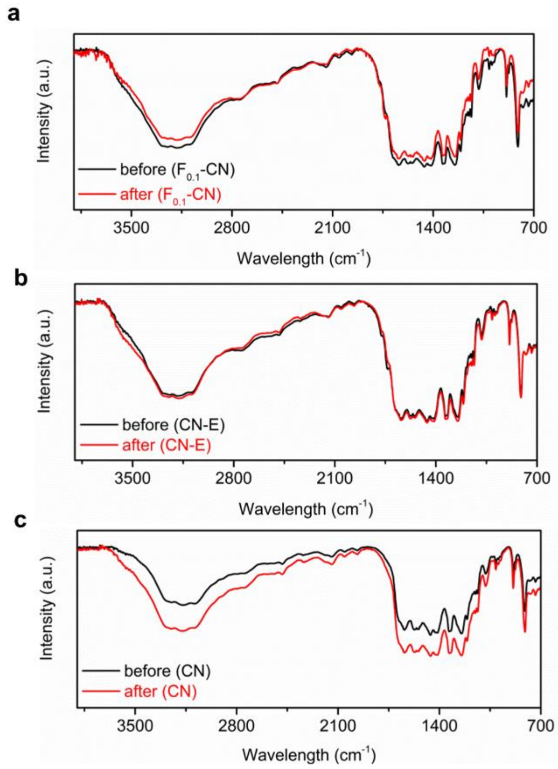

Supplementary Figure 7. Control experiments show the reaction stability of the sample. The comparison of DRIFTS spectra on **(a)** $F_{0.1}$-CN, **(b)** CN-E, and **(c)** CN samples before and after 5 hrs of reaction.

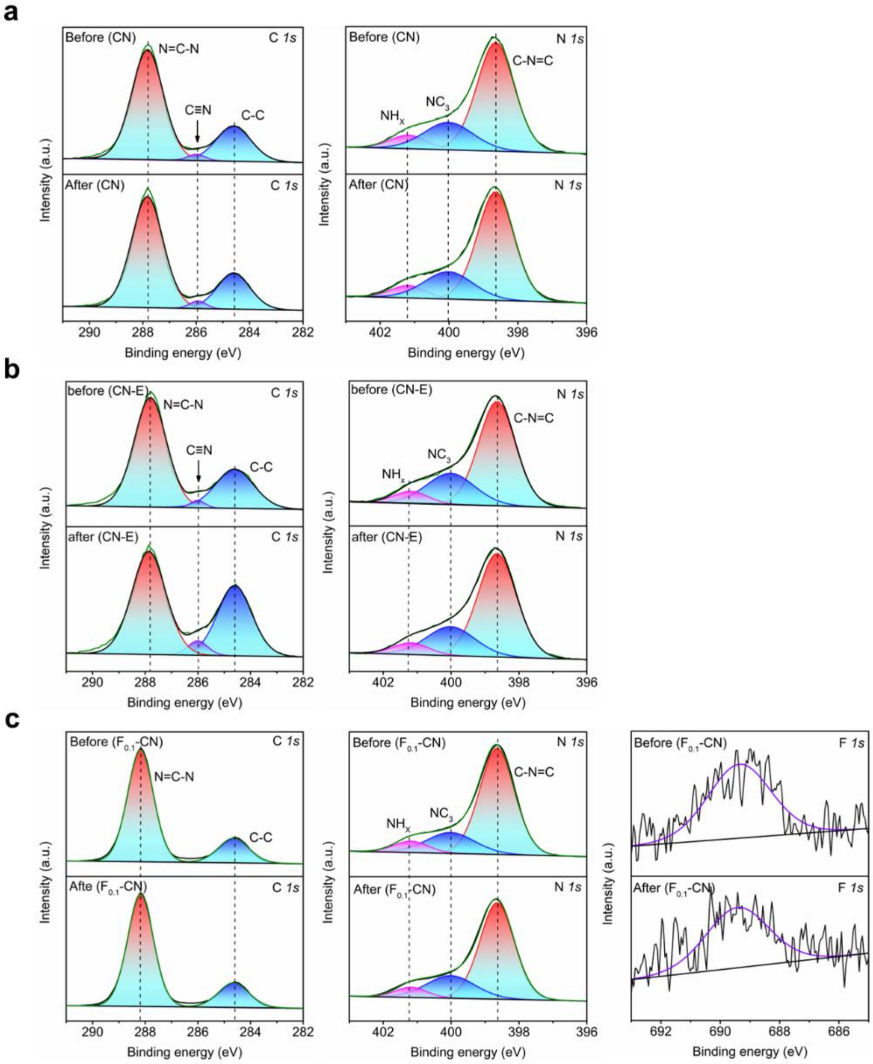

Supplementary Figure 8. Control experiments show unchanged electronic states after the reaction. The comparison of XPS spectra on **(a)** CN (C*1s* and N*1s*), **(b)** CN-E (C*1s* and N*1s*), and **(c)** $F_{0.1}$-CN (C*1s*, N*1s*, and F*1s*) samples before and after 5 hrs of reaction.

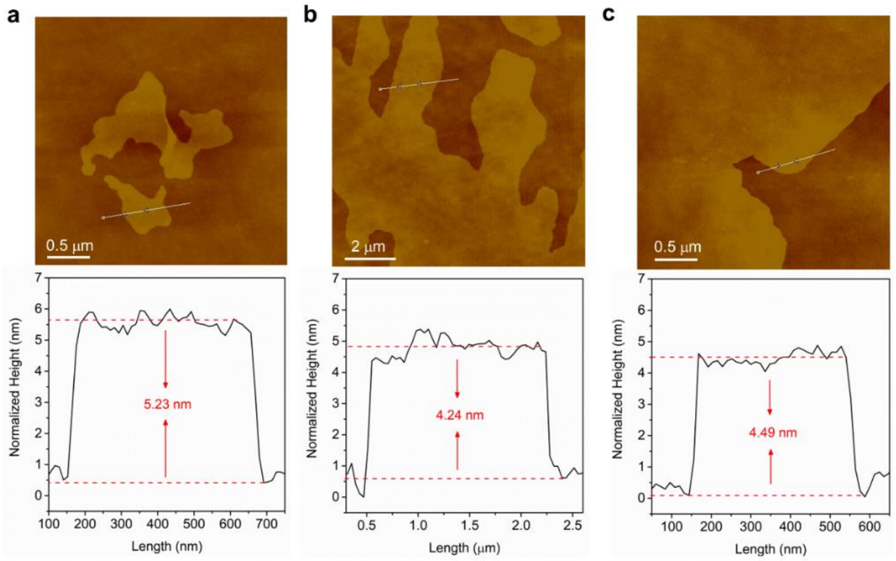

Supplementary Figure 9. The determination of sample thickness. AFM images and corresponding line scan to determine the thickness of **(a)** CN, **(b)** CN-E, and **(c)** $F_{0.1}$-CN.

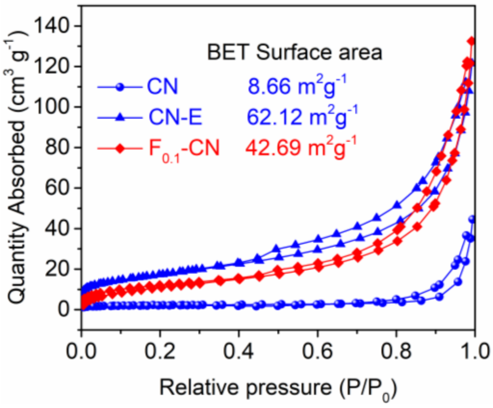

Supplementary Figure 10. Comparison of BET surface area of different samples. N_2 adsorption-desorption isotherms of CN, CN-E and $F_{0.1}$-CN. inset shows the BET surface area of different samples.

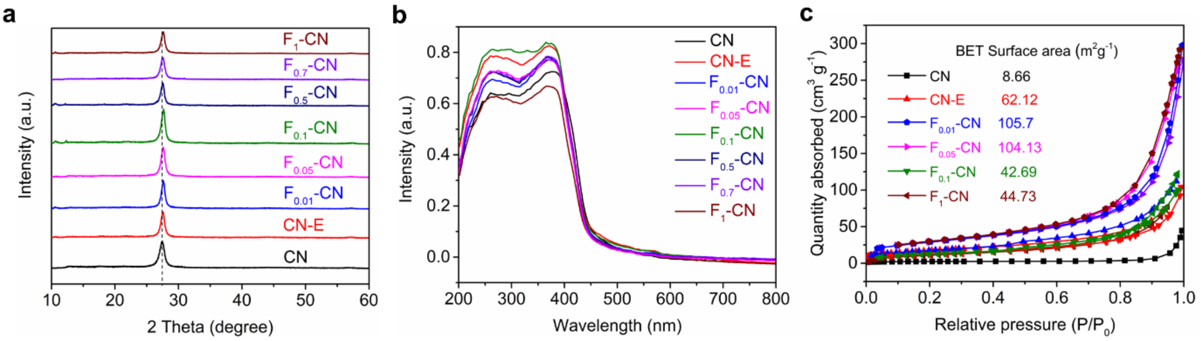

Supplementary Figure 11. Control experiments to show the effect of F content. (a) XRD patterns, **(b)** UV-vis DRS spectra, and **(c)** N_2 adsorption-desorption isotherms of CN, CN-E and F-CN samples with different F contents. Inset of **(c)** shows the BET surface area of different samples

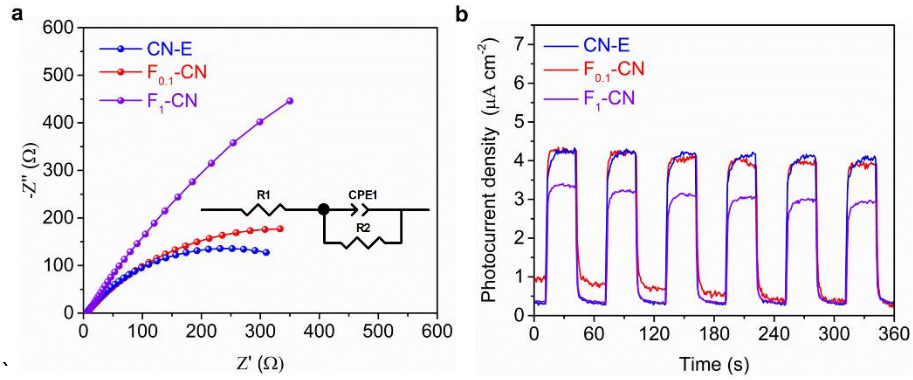

Supplementary Figure 12. Electrochemical characterizations to show the effect of F-modification. (a) EIS and **(b)** photocurrent curves of CN-E, $F_{0.1}$-CN, and F_1-CN sample.

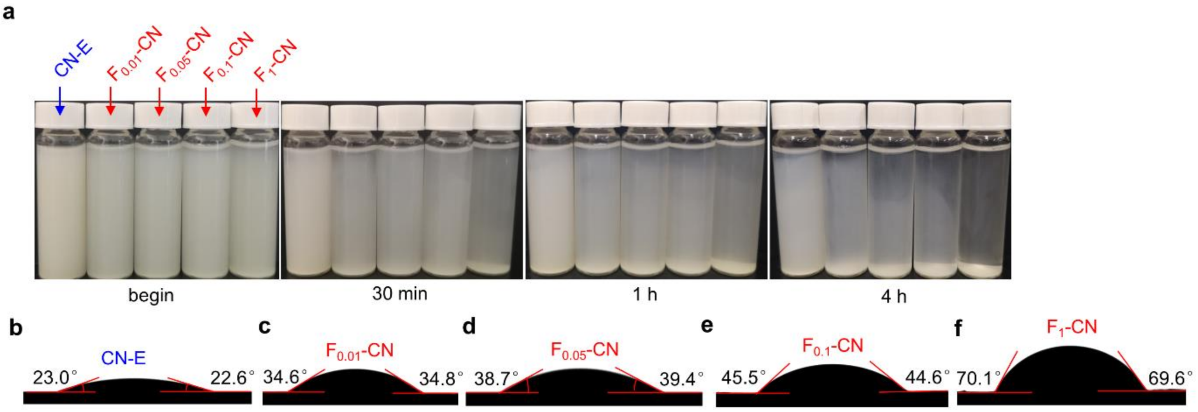

Supplementary Figure 13. Control experiments to show the effect of F-content on surface wettability. (a) The comparison of continuous sedimentation of CN-E and F-CN samples with different F content, and water surface contact angle on **(b)** CN-E, **(c)** $F_{0.01}$-CN, **(d)** $F_{0.05}$-CN, **(e)** $F_{0.1}$-CN, and **(f)** F_1-CN.

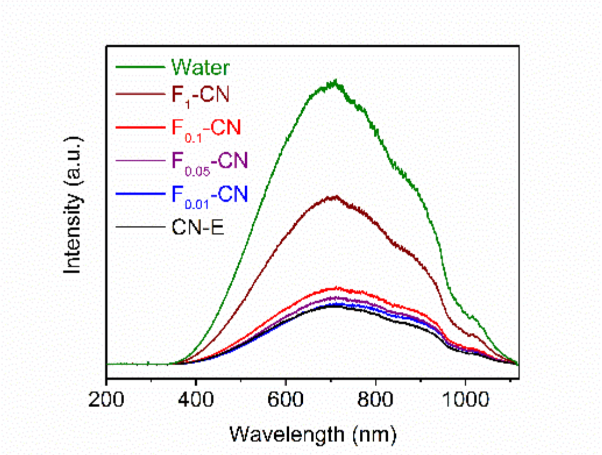

Supplementary Figure 14. Control experiments to show increased light transmission with high F-content. Light transmittance through different catalyst suspensions (0.3g/L) by in situ optical fiber spectroscopy (under constant 500 rpm magnetic stirring). The white light source is a tungsten lamp (5W). Water is the transparent solvent.

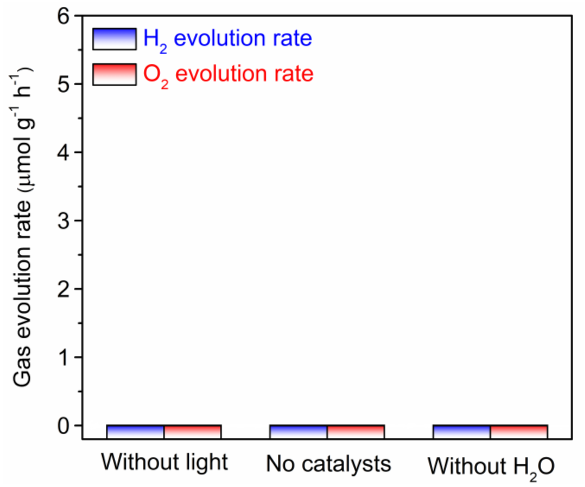

Supplementary Figure 15. Control experiments to confirm photocatalytic reaction. Photocatalytic H_2 and O_2 production without light, without catalysts or without H_2O on $F_{0.1}$-CN sample.

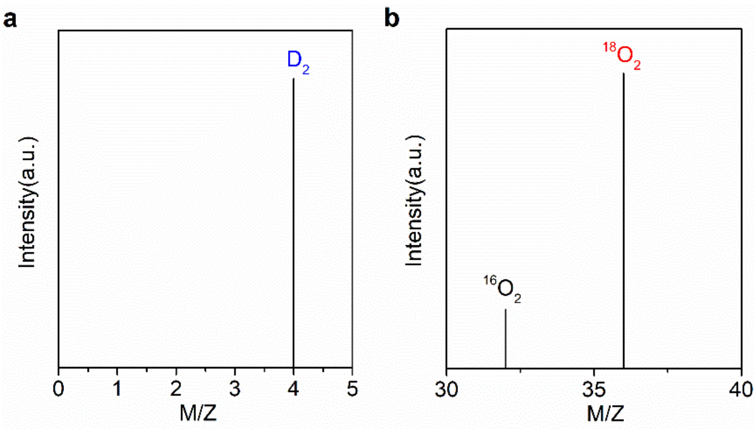

Supplementary Figure 16. Isotopically labeled experiments to show products from water splitting. GC-MS profiles of gas products with $F_{0.1}$-CN catalyst in **(a)** deuterium-labeled D_2O and **(b)** ^{18}O-labeled $H_2{}^{18}O$.

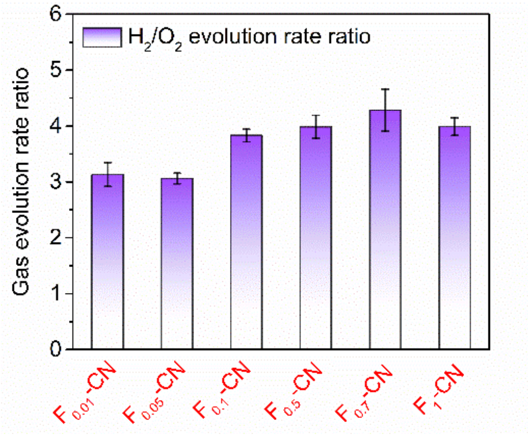

Supplementary Figure 17. Comparison of H_2/O_2 ratio with different F-contents. The H_2/O_2 evolution rate ratio profile on different F-CN catalysts. Error bar Error bars were obtained by statistically repeating identical experimental results three times.

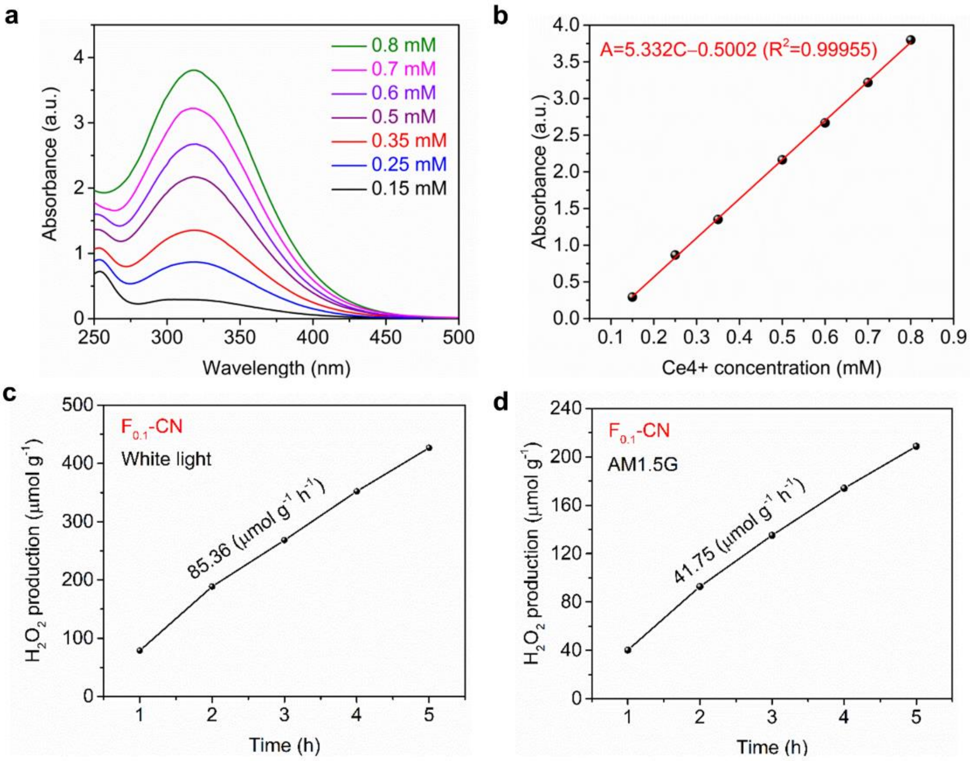

Supplementary Figure 18. Quantification of H_2O_2 production. (a) UV-vis absorption spectra of Ce^{4+} solution in different concentrations (0.15 mM-0.8 mM); **(b)** The concentration standard curve of Ce^{4+} solution. **(c)** The white-light photocatalytic H_2O_2 production and **(d)** AM1.5G irradiation H_2O_2 production profiles on $F_{0.1}$-CN determined by the back titration of Ce^{4+}.

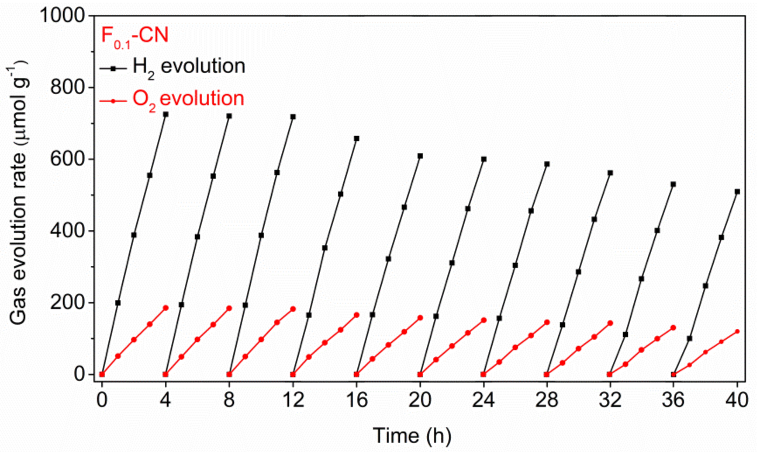

Supplementary Figure 19. Long-term reaction stability experiments. Time course of the white-light photocatalytic overall water splitting on $F_{0.1}$-CN in 40 hrs.

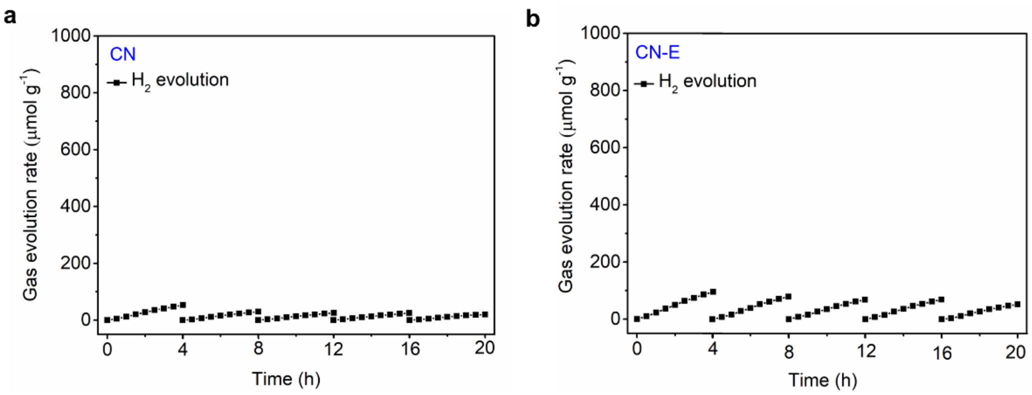

Supplementary Figure 20. Long-term reaction stability experiments on inert catalysts. Time course comparison of the white-light photocatalytic overall water splitting on CN (a) and CN-E (b) catalysts.

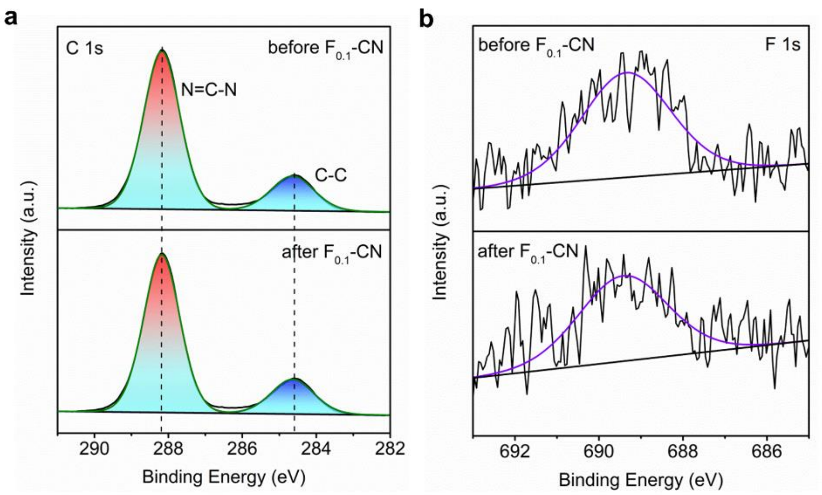

Supplementary Figure 21. Control experiments to show the stability of C-F bond. The comparison of XPS **(a)** C*1s* and **(b)** F*1s* spectra on $F_{0.1}$-CN before and after 5 hrs of reaction.

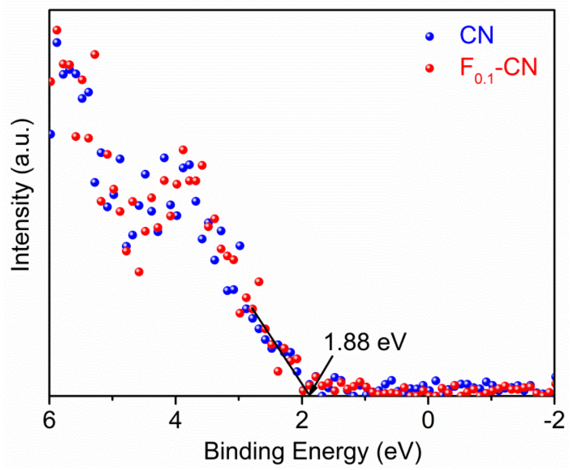

Supplementary Figure 22. Determination of valence band position. XPS valence band spectra near Fermi level of the CN and $F_{0.1}$-CN samples.

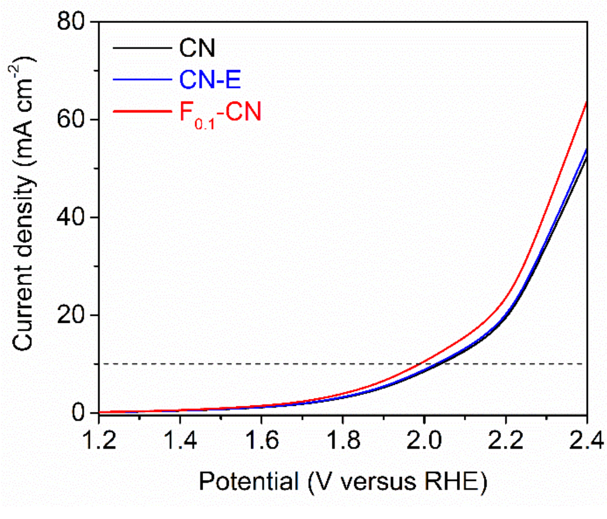

Supplementary Figure 23. Comparison of electrochemical OER potentials. Polarization curves of CN, CN-E and $F_{0.1}$-CN samples.

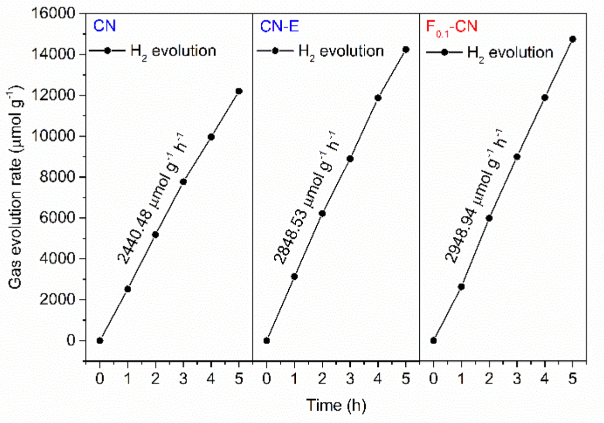

Supplementary Figure 24. Comparison of HER activities. The H_2 production profiles on CN, CN-E, and F0.1-CN catalysts with triethanolamine as the hole scavenger.

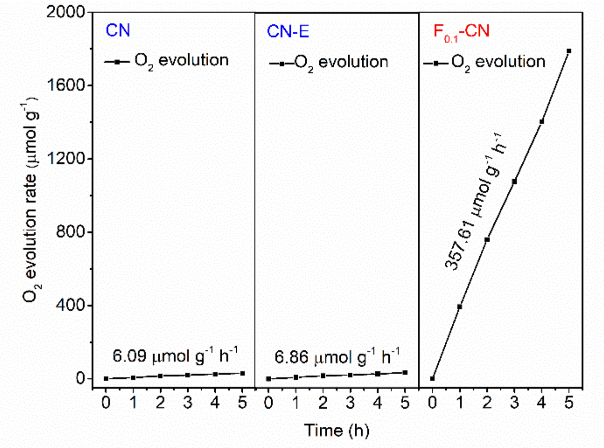

Supplementary Figure 25. Comparison of OER activities. OER profiles on **(a)** CN and **(b)** CN-E, and **(c)** $F_{0.1}$-CN catalysts with 1M $AgNO_3$ as electron acceptor under white light irradiation.

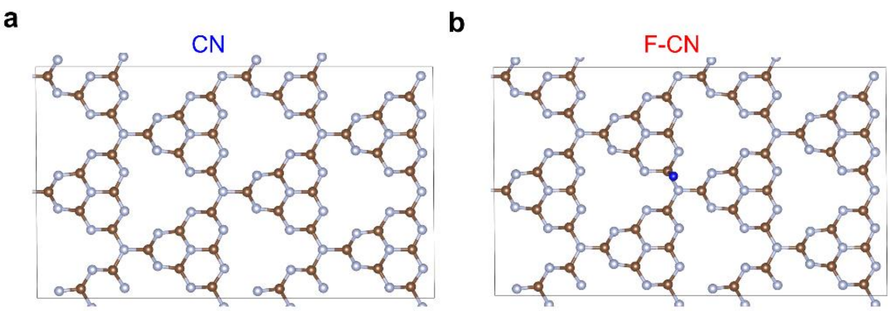

Supplementary Figure 26. Structural diagrams of CN and F-CN. Simulated 2 × 3 supercell of (a) CN and (b) F-CN layers by using one F atom to bond with the C atom in CN.

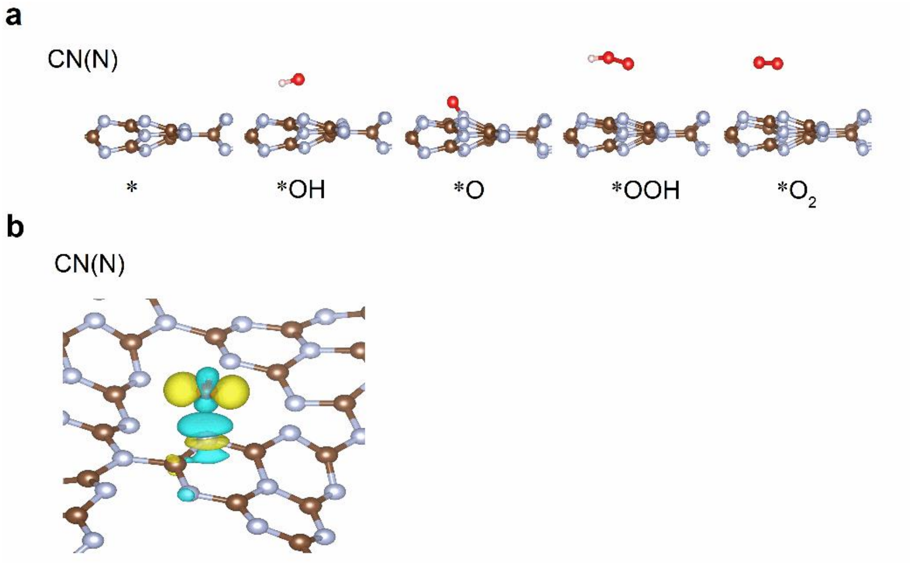

Supplementary Figure 27. Simulated OER pathway on CN(N). (a) Water adsorption and activation simulation on N site in pristine CN (denoted CN(N)). **(b)** Charge density difference mappings between *OH intermediate and catalyst surface on the CN(N). The blue and yellow isosurfaces stand for the negative and positive charges, respectively.

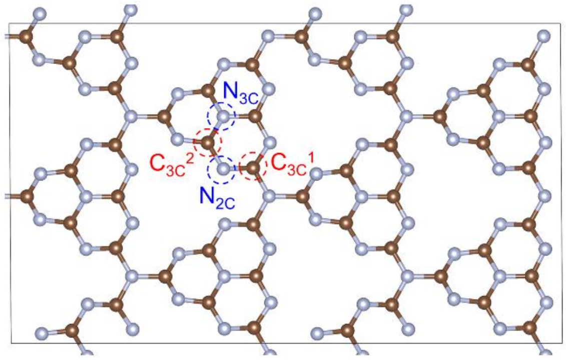

Supplementary Figure 28. The diagram shows different carbon sites on CN. Simulated structural configuration of CN supercell (2 × 3).

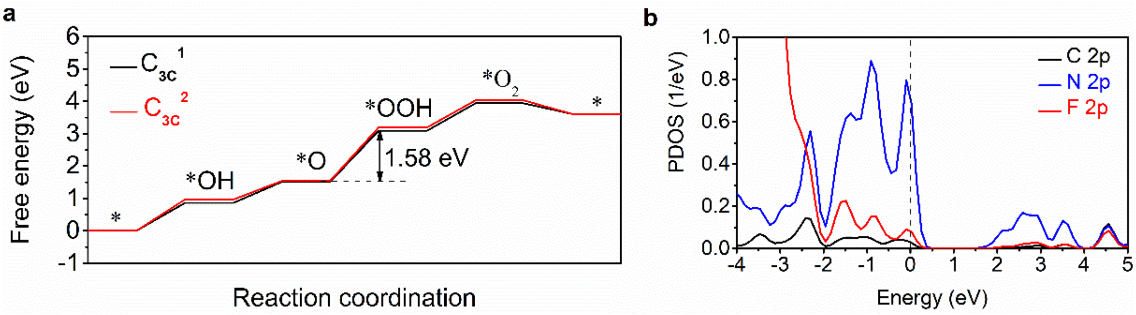

Supplementary Figure 29. DFT simulations on OER steps with different carbon sites occupied. (a) Free energy profiles of OER on F-CN at pH = 7 and U = 0 V vs SHE (where * represents the intermediate state). $C_{3c}1$ profile represents N reaction sites with neighbored $C_{3c}1$ site occupied by F atom; $C_{3c}2$ profile represents N reaction sites with neighbored $C_{3c}2$ site occupied by F atom; **(b)** PDOS of 2p states of surface C, N and F in F-CN with C_{3c}^2 occupied by F atom. The dashed line stands for Fermi level.

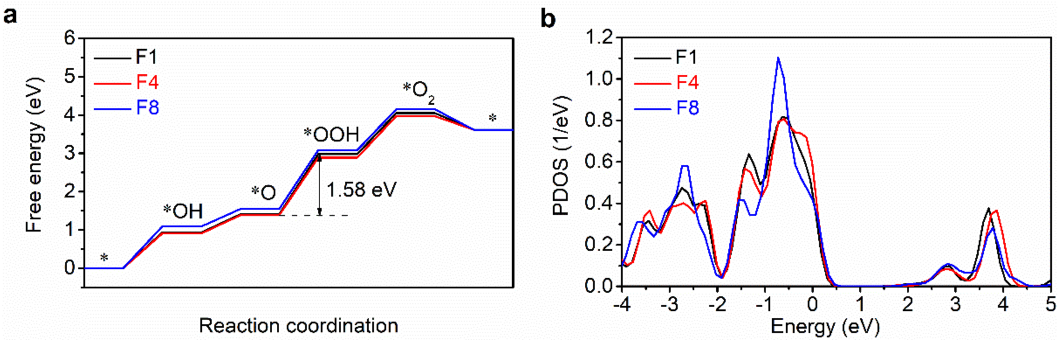

Supplementary Figure 30. DFT simulations with F-content. (a) Free energy profiles of OER on F-CN(N) with F surface coverage of 1 atom% to 8 atom% (denoted F1~F8). F-CN(N) represents N reaction sites on F-CN (C reaction sites occupied entirely by F atoms). **(b)** PDOS of N *2p* states in F-CN with F surface coverage of 1 atom% to 8 atom% (denoted F1~F8). The dashed line stands for Fermi level.

Supplementary Tables

Supplementary Table 1. Summary of detailed results of the NAP-XPS fit parameters.

Sample	C-O(530.1 eV)	O-H(531.3eV)	C=O(532.7eV)	Test conditions
CN	11489.16	10983.41	-	Pristine
CN	10279.90	10348.48	-	Light 0min+0.2mbar H_2O
CN	10762	10087.80	9667.29	Light 5min+0.2mbar H_2O
CN	10396.01	10073.63	9636.29	Light 10min+0.2mbar H_2O
CN	9529.22	8910.69	8687.83	Light 15min+0.2mbar H_2O
$F_{0.1}$-CN	12530.89	12337.01	-	Pristine
$F_{0.1}$-CN	10940.31	10599.66	-	Light 0min+0.2mbar H_2O
$F_{0.1}$-CN	12850.41	12381.24	-	Light 5min+0.2mbar H_2O
$F_{0.1}$-CN	11156.77	10862.88	-	Light 10min+0.2mbar H_2O
$F_{0.1}$-CN	12067.84	11951.31	-	Light 15min+0.2mbar H_2O

Sample	C-N=C(398.3eV)	NC_3(399.1eV)	NH_X(400.3eV)	Test conditions
CN	13486.99	12226.83	12073.94	Pristine
CN	12373.09	11354.84	11147.84	Light 0min+0.2mbar H_2O
CN	11746.45	11358.30	10975.78	Light 5min+0.2mbar H_2O
CN	11608.07	11192.55	10816.01	Light 10min+0.2mbar H_2O
CN	11180.82	10802.65	10494.91	Light 15min+0.2mbar H_2O
$F_{0.1}$-CN	14549.46	14068.37	13692.77	Pristine
$F_{0.1}$-CN	14206.92	13148.05	13106.73	Light 0min+0.2mbar H_2O
$F_{0.1}$-CN	12525.98	11180.03	11649.44	Light 5min+0.2mbar H_2O
$F_{0.1}$-CN	13807.75	13376.22	13047.36	Light 10min+0.2mbar H_2O
$F_{0.1}$-CN	12663.65	12279.95	11900.27	Light 15min+0.2mbar H_2O

CN / Specie	Position(eV)	Integral area	Test conditions
C-C	284.4	19554.74	Pristine
C-C	284.5	11939.11	Light 0min+0.2mbar H_2O
C-C	284.7	11242.94	Light 5min+0.2mbar H_2O
C-C	284.8	11847.86	Light 10min+0.2mbar H_2O
C-C	285	13261.09	Light 15min+0.2mbar H_2O
N=C-N	287.7	19645	Pristine
N=C-N	287.8	12981.81	Light 0min+0.2mbar H_2O
N=C-N	288	12139.18	Light 5min+0.2mbar H_2O
N=C-N	288.1	12787	Light 10min+0.2mbar H_2O
N=C-N	288.3	13895	Light 15min+0.2mbar H_2O

$F_{0.1}$-CN / Specie	Position(eV)	Integral area	Test conditions
C-C	284.6	15848.52	Pristine
C-C	284.7	12973.31	Light 0min+0.2mbar H_2O
C-C	284.7	16903.55	Light 5min+0.2mbar H_2O
C-C	284.7	15140.01	Light 10min+0.2mbar H_2O
C-C	284.7	14902.10	Light 15min+0.2mbar H_2O
N=C-N	288.1	16681.32	Pristine
N=C-N	288.1	14008.79	Light 0min+0.2mbar H_2O
N=C-N	288.1	17326.03	Light 5min+0.2mbar H_2O
N=C-N	288.2	16069.74	Light 10min+0.2mbar H_2O
N=C-N	288.2	15889.67	Light 15min+0.2mbar H_2O

Supplementary Table 2. Elemental analysis of CN, CN-E, and F-CN samples.

Sample	C/N (at%)	F^- (mg/Kg)	F/C (at%)	F/C_{surf} (at%)
CN	64.98	0	0	0
CN-E	65	0	0	0
$F_{0.01}$-CN	65.08	1288.1939	0.2753	1.7895
$F_{0.05}$-CN	65.22	1617.9717	0.2854	1.8551
$F_{0.1}$-CN	65.24	3547.5642	0.6270	4.0755
$F_{0.5}$-CN	65.29	5718.8241	1.0216	6.6404
$F_{0.7}$-CN	65.47	13684.4692	2.4370	15.8405
F_1-CN	65.52	61008.9655	11.4104	74.1676

Supplementary Table 3. Wavelength-dependent water splitting performances and AQYs on $F_{0.1}$-CN catalyst.

Wavelength (nm)	365	380	405	420	435	450	475	500
H_2 evolution (μmol)	6.3127	5.3863	2.8891	2.1345	0.4584	0.2695	0	0
O_2 evolution (μmol)	1.5782	1.4879	0.7408	0.5431	0.1161	0.0842	0	0
Light intensity (mW cm^{-2})	10.06	11.64	101.3	102.9	97.6	105.1	78.6	96.9
Irradiation area (cm^2)	4	4	4	4	4	4	4	4
Irradiation time (h)	5	5	5	5	5	5	5	5
AQY (%)	0.5718	0.3538	0.023	0.0164	0.0036	0.0019	0	0

Supplementary Table 4. Wavelength-dependent water splitting performances and AQYs on the CN catalyst.

Wavelength (nm)	365	380	405	420	435	450	475	500
H_2 evolution (μmol)	1.6081	1.0457	0.1352	0.0678	0	0	0	0
O_2 evolution (μmol)	0	0	0	0	0	0	0	0
Light intensity (mW cm^{-2})	11.45	12.75	102.4	103.5	96.8	113.1	77.3	97.4
Irradiation area (cm^2)	4	4	4	4	4	4	4	4
Irradiation time (h)	5	5	5	5	5	5	5	5
AQY (%)	0.1280	0.0718	0.0011	0.0005	0	0	0	0

Reaction conditions: 30 mg catalysts, 100 ml deionized water, using 3wt% Pt as co-catalysts without any sacrificial agents.

All reactions were carried out at 279 K in low vacuum.

Take λ=420 nm of the $F_{0.1}$-CN as example:

The number of incident photons:

$$\mathrm{N} = \frac{E\lambda}{hc} = \frac{102.9\times4\times10^{-3}\times5\times3600\times420\times10^{-9}}{6.626\times10^{-34}\times3\times10^{8}} = 1.5654\times10^{22} \qquad (5)$$

AQY:

$$\mathrm{AQY} = \frac{2\times\text{the number of evolved } \mathrm{H_2} \text{ molecules}}{\mathrm{N}}\times100\% \qquad (6)$$

$$= \frac{2\times6.02\times10^{23}\times2.1345\times10^{-6}}{1.5654\times10^{22}}\times100\% = 0.0164\,\%$$

Supplementary Table 5. Calculated energies of different intermediate state in respect to the initial * state (0 eV).

	∗ (eV)	∗OH (eV)	∗O (eV)	∗OOH (eV)	$*O_2$ (eV)	∗ (eV)
CN (C)	0	2.25	3.10	5.00	4.03	3.61
CN (N)	0	2.86	2.00	4.24	4.08	3.61
F-CN (N)	0	0.86	1.51	3.09	3.94	3.61
